# Therapeutic Value of Hydrate of Chloral in Certain Forms of Convulsive Disorder

**Published:** 1877-09-20

**Authors:** Charles A. Rayne


### THERAPEUTIC VALUE OF HYDRATE OF CHLORAL IN CERTAIN FORMS OF CONVULSIVE DISORDER.

BY DR. CHARLES A. RAYNE.

In the number of the *Lancet* fot March 13th, 1875, I reported a case of convulsions in a child under the care of Dr. Acland, at the Radcliffe Infirmary, Oxford, which readily yielded to treatment by hydrate of chloral after failure of the usual remedies, and in which the cure, so far as could be afterwards discovered, was complete and permanent.

The case presented the following distinctive features: The patient, a boy aged five, was of lively and intelligent disposition, and of good general health. The attacks followed, and were attributed to a blow upon the head, though it was discovered that entozoa were present in the intestine, the removal of which, however, failed to have more than an incomplete and temporary effect in the cure of the disorder. The convulsions were very frequent, often numbering twenty to thirty in twenty-four hours, but short in duration, and singularly abrupt and sudden, both in onset and termination. They occurred both night and day, and were attended with absolute loss of con sciousness, and with muscular phenom ena which corresponded very closely with such as were artificially obtained by Professor Ferrier on stimulation of certain frontal and parietal convolutions of some of the lower animals.

I have recently had under my care, at the Manchester General Hospital for Children, a case which presents many features in common with the above, and which also yielded to hydrate of chloral treatment after the failure of other remedies.

J. A., a girl aged nine, was admitted on May 23d, with convulsive attacks. These commenced a year ago, without known cause, and occurred then to the number of three or four in the day. They disappeared under medical treatment in the course of three months, but reappeared about six months ago with much increased frequency, and though she has since this time been under the care in succession of several medical men in the town, there has been no improvement. Her mother was subject to "fits" three years ago, in which she fell down and lost her consciousness, these occurring two or three times in the day, but disappearing at the end of four months. The rest of the family, which is a large one, are healthy.

The patient is a healthy-looking, well-nourished girl, quick and intelligent in her general appearance, and in her answers to inquiries. There is no affection of the heart or kidneys discoverable, nor reason to suspect the presence of entozoa, or other sources of internal irritation. The attacks number about fifteen in the twenty-four hours, and occur both night and day, at pretty regular intervals of one to two hours. Several were carefully watched. There is not the slightest aura of any description, the fit coming on at once without any warning in the middle of any occupation she may be engaged in. The onset is so sudden that it is impossible to decide what group of muscles is first affected. The attack is limited chiefly to the upper part of the body, and is not unilateral. The head is drawn down, and both sterno mastoids are rigid. Both arms become rigid in semi extension, and are seized with a tremulous motion. The facial muscles are unaffected, so far as can be seen; the eye-balls are motionless and straight, the pupils being somewhat dilated and conjunctivæ insensible. A groaning kind of noise is made during the time the fit lasts (about one minute), respiration being labored and rapid, and apparently taking place through a contracted glottis. Recovery is rapid and complete, there being no after drowsiness, headache, or intellectual confufusion. From examination during the attack, as well as a careful questioning of the patient, it appears that consciousness is entirely suspended during the attack.

Patient was at first kept quietly in bed without medicine, with the view of testing the effect of a regulated condition of living. There being no improvement, on the 26th bromide of potassium, in five-grain doses, thrice daily, was ordered; on the 29th, ten-grain doses of the medicine, thrice daily, were given, and on the 30th one drachm at a single dose. This treatment had not the slightest effect of any kind; the attacks remained the same in character and duration, and still numbered abont fifteen in the twenty-four hours. Tincture of belladona was then given in five minim doses every six hours, and was increased on June 1st to eight minims. The fits now numbered from fourteen to eighteen in twenty-four hours, and were unaltered in character and duration.

June 3. Patient took eight grains of chloral hydrate at 5 P. M., and had no fits during the next seven hours (about the length of time in myself of a sleep produced by the drug, and which perhaps is connected with its period of elimination from the body). There was no sleepiness after the chloral. From midnight to 10 A. M. there were seven fits. Always sleeps well at night.

5th. There has been no further dose of chloral given, and the patient has had in the last twenty-four hours twenty-three fits, which brings up the average for the two days to fifteen again. Ordered five grains of the chloral twice daily, at 10 A.M. and 4 P.M.

8th. Has had five fits each day, and these occurred always during the night hours, from 11 P.M. to 5 A.M., except on the 7th, when there was a fit in the day time, evidently due to disturbance caused by a small dose (one drachm) of castor oil, for it occurred half an hour afterwards, and on this day the fits numbered seven in the day. Ordered five grains every six hours, commencing at 10 A. M.

12th. Has had from three to four fits each day, all occurring in the early morning hours, from 1 to 6 A.M. The night doses at 10 P. M. and 4 A. M., ordered to be increased to ten grains. Al ways sleeps well at night, notwithstanding the fits.

21st. Up to the 16th patient had one to two fits each night, but since this time there have been none. Ordered five grains of chloral at 10 P.M. and 4 A.M.; none during the day.

25th. There has been no fit; ordered to stop the chloral altogether.

July 10th. There has been no return of the fits since June 16th, and patient is discharged apparently in every way healthy.—*Lancet.*

